# Implications of spatially heterogeneous vaccination coverage for the risk of congenital rubella syndrome in South Africa

**DOI:** 10.1098/rsif.2012.0756

**Published:** 2013-01-06

**Authors:** C. J. E. Metcalf, C. Cohen, J. Lessler, J. M. McAnerney, G. M. Ntshoe, A. Puren, P. Klepac, A. Tatem, B. T. Grenfell, O. N. Bjørnstad

**Affiliations:** 1 Department of Zoology, Oxford University, Oxford, UK; 2 Fogarty International Center, National Institute of Health, Bethesda, MD, USA; 3 Centre for Respiratory Disease and Meningitis, National Institute for Communicable Diseases, National Health Laboratory Service, Johannesburg, South Africa; 4 School of Public Health, Faculty of Health Sciences, University of the Witwatersrand, Johannesburg, South Africa; 5 Department of Epidemiology, John Hopkins Bloomberg School of Public Health, Baltimore, MA, USA; 6 Centre for Vaccines and Immunology, National Institute for Communicable Diseases, National Health Laboratory Service, Johannesburg, South Africa; 7 Division of Virology and Communicable Diseases, School of Pathology, Faculty of Health Sciences, University of the Witwatersrand, Johannesburg, South Africa; 8 Department of Ecology and Evolutionary Biology, Princeton University, Eno Hall, Princeton, NJ, USA; 9 Department of Geography, and Emerging Pathogens Institute, University of Florida, Gainesville, FL, USA; 10 Centre for Infectious Disease Dynamics, The Pennsylvania State University, 208 Mueller Lab, University Park, PA, USA

**Keywords:** rubella, vaccination, spatial variation

## Abstract

Rubella is generally a mild childhood disease, but infection during early pregnancy may cause spontaneous abortion or congenital rubella syndrome (CRS), which may entail a variety of birth defects. Since vaccination at levels short of those necessary to achieve eradication may increase the average age of infection, and thus potentially the CRS burden, introduction of the vaccine has been limited to contexts where coverage is high. Recent work suggests that spatial heterogeneity in coverage should also be a focus of concern. Here, we use a detailed dataset from South Africa to explore the implications of heterogeneous vaccination for the burden of CRS, introducing realistic vaccination scenarios based on reported levels of measles vaccine coverage. Our results highlight the potential impact of country-wide reductions of incidence of rubella on the local CRS burdens in districts with small population sizes. However, simulations indicate that if rubella vaccination is introduced with coverage reflecting current estimates for measles coverage in South Africa, the burden of CRS is likely to be reduced overall over a 30 year time horizon by a factor of 3, despite the fact that this coverage is lower than the traditional 80 per cent rule of thumb for vaccine introduction, probably owing to a combination of relatively low birth and transmission rates. We conclude by discussing the likely impact of private-sector vaccination.

## 1. Introduction

Rubella is a mild infection if contracted during childhood, but infection during early pregnancy can lead to birth of a child with congenital rubella syndrome (CRS), entailing numerous potential disabilities with substantial financial and social costs [1,2]. Although a completely immunizing, safe and relatively cheap vaccine exists, the possibility that inadequate vaccination coverage may increase the CRS burden by raising the average age of infection without sufficiently decreasing transmission (a pattern suggested to have occurred in Greece [3] and Costa Rica [4]) has led to considerable caution in its introduction. This caution may be meretricious, but it may also result in a missed opportunity because the rubella vaccine is easily combined with the measles vaccine, and the current enhanced efforts at measles control could potentially provide an effective vehicle for, at least, local elimination of rubella [5]. However, unless control measures are synchronized within a state or a broader region, there is a risk that the benefits of reduced disease incidence will be inequitably distributed, and potentially even worsened in some communities. The reason for this risk is that spatial heterogeneity in vaccine coverage may lead to broken chains of transmission and transient local elimination of rubella in certain areas [6]. Women in these communities may then remain susceptible to rubella into their childbearing years. Exposure through rare contacts with infected individuals from communities in which vaccine coverage is low enough to allow rubella to circulate could then occur, thus enhancing the CRS rate. In this way, age-specific risk of a disease is affected by vaccine-induced heterogeneities in circulation, operating either via spatial heterogeneities in vaccine cover or via homogeneous vaccine cover of sufficient magnitude to break the chains of transmission and push communities below the critical community size (e.g. the population size above which immunizing childhood infections are not vulnerable to stochastic extinction [7]).

Here, we use a uniquely detailed spatio-temporal dataset from South Africa to explore both the basic epidemiology of the infection, and the repercussions likely to follow the introduction of a rubella-containing vaccine, using recent measles coverage as a template. Rubella vaccination has not been introduced into the public sector in South Africa, but incidence data are available via measles surveillance activities. In addition to estimating rubella transmission rates and contact patterns, a key question addressed here is whether spatial variation in vaccine coverage (as reported in South Africa for measles [8,9]) is likely to lead to increases in the CRS burden at either global or local scales. Specifically, we explore what spatial patterns of vaccination might inadvertently favour metapopulation rescue effects (i.e. re-introduction of the infection into districts where it has gone locally extinct) developing methods to test for the effect of the link between connectivity, coverage and population size. We then assess whether observed measles coverage levels are likely to result in global or local increases in the CRS burden.

## 2. Material and methods

### 2.1. Data

The data on laboratory-confirmed cases of rubella were obtained from the South African National Institute for Communicable Diseases, a division of the National Health Laboratory Service (NICD-NHLS). Specimens were submitted as part of national, active, case-based measles surveillance. In South Africa, measles is a notifiable disease, and all patients meeting the suspected-measles case definition (rash and fever with at least one of cough, coryza or conjunctivitis) should have specimens taken. Blood and throat/nasopharyngeal swab or urine specimens are sent on ice to the NICD-NHLS for laboratory confirmation where testing is conducted at no charge. All serum specimens from suspected-measles cases were tested for the presence of rubella-specific immunoglobin antibodies, using an enzyme-linked immunosorbent assay (ELISA; Enzygnost, Siemens, Marburg, Germany).

District-level population sizes were obtained from Statistics South Africa and birth rates through adjusting census microdata (https://www.international.ipums.org/international/) on numbers of infants with subnational data on infant mortality rates [10]. Districts range in population size from less than 100 000 (three districts in the Northern and Western Cape) to more than 3 million (for the three districts with capitals Durban, Cape Town and Johannesburg). District-scale measles vaccination coverage of 1 year olds (inferred from the number of doses given among children less than 1 year old) from 2000 to 2010 was obtained from the South African National Department of Health Expanded Programme on Immunization (J. van den Heever 2011, personal communication). This was used to simulate spatially variable rubella vaccination coverage over the time-frame for which incidence data were available; years prior to 2000 were assumed to have the same coverage as 2000. To analyse spatial connectivity between districts, matrices describing the distance between each of the districts and the cost of travelling between each of the districts were obtained [11]. To estimate the cost of travelling, in each case, population-adjusted district centroids were calculated, using gridded population data (www.afripop.org) to locate a ‘centroid’ for each district at the settlement of greatest population size. The great circle distances between each centroid and every other centroid were calculated, as well as the ‘accessibility’ distance, calculated using land-use data to represent the ease of movement across a realistic landscape [11,12].

### 2.2. Fitting the time-series susceptible–infected–recovered model

To estimate the overall rate of transmission, averaging across the age-heterogeneities, we use the time-series susceptible–infected–recovered (TSIR) model. A widespread issue for the analysis of infectious disease dynamics is under-reporting, resulting in the total number of infected individuals being unknown. However, reporting rates can be indirectly estimated. Here, we consider the dynamics of total number of susceptible individuals, *S_t_*, and infected individuals, *I_t_*, at time *t*. If reporting rates are stable through time, and all individuals eventually succumb to infection, numbers of susceptibles in any given location will track local births, *B_t_*, and infected individuals. The balance equation for susceptible individuals is2.1
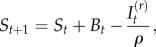


where *ρ* is the reporting rate, and 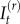 is the reported number of infected cases. Ignoring observational uncertainty, the actual number of infected individuals can be reconstructed as 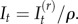 Rearranging equation (2.1) provides the relationship from which the reporting rate and the dynamics of the susceptible population can be inferred through susceptible reconstruction [13,14]:2.2
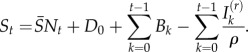


In equation (2.2), 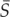 represents the average proportion of individuals that are susceptible, *N_t_* represents the population size and *D*_0_ is the unknown deviation around the average at the time of the first observation in the time-series. To estimate *ρ* and reconstruct a full time-series of susceptible ‘deviations’, *D_t_*, that details how the numbers of susceptible individuals vary around the average number of susceptible individuals, we write2.3
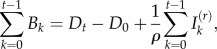


where 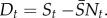 From this, *D_t_* can be estimated as the residuals from the possibly locally varying regression of the cumulative number of births on the cumulative number of cases, and *ρ* can be estimated as the inverse slope of this regression [13,15]. Note that the average number of susceptible individuals cannot be directly estimated, as it is confounded with the intercept of this regression equation.

Armed with the time-series *I_t_* and *D_t_*, seasonal transmission rates can be estimated [13,15]. The generation time (serial interval) of rubella (approximately the latent plus infectious period) is approximately 18 days [16,17]; so we assumed that the time-scale of the epidemic process was approximately two weeks, and aggregated the data accordingly. The number of infected individuals at time *t* + 1; depends stochastically on *I_t_* and *S_t_* with expectation 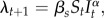 where *β_s_* is the seasonally varying age-averaged transmission rate and *s* denotes the season. The exponent *α*, usually a little less than 1, captures heterogeneities in mixing not directly modelled by the seasonality [13,15] and the effects of discretization of the underlying continuous time process [18]. Then, taking logs on both sides of the relationship for the expectation, we can write2.4
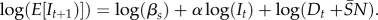


Given estimates of *I_t_* and *D_t_*, regression can be used to estimate *β_s_*. We use the profile of the likelihood estimated across a range of values of the proportion susceptible to estimate 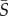 [14,15]. We have previously found that low reporting rates result in strongly downwards-biased estimates of *α*, which result in unrealistic dynamics. We previously proposed a Whittle estimator to correct for this [19]. However, that approach requires very long time-series. In this study, we therefore fix *α* at a consensus value of 0.97 [15,18,20] and estimated seasonal transmission as above. The transmission rate estimated in this way may reflect a broad range of processes that occur consistently over the course of a year. For childhood infections, low transmission usually mirrors periods of school vacation, indicating that the parameter captures mixing among school children [19]; for other infections, climatic variables such as absolute humidity (e.g. influenza, [21]) may be more important.

Transmission of childhood infections in both industrialized and pre-industrialized countries usually scales in a frequency-dependent fashion, because social clique size is relatively constant [15,19,22]. Therefore, it is convenient to consider the alternative parametrization of the TSIR, where λ*_t_* = *β*‘ *S_t_ I_t_*/*N*, where *β*‘ *= *β* N*. Because standard errors on district estimates were considerable due to low local incidence, we used the South-Africa-wide estimate of seasonality in transmission, and adjusted the median value for each location by the ratio between the local population size, and the size of the entire population of South Africa [20]. Note that although the number of years available in this dataset is small relative to previous analyses (e.g. in England and Wales, 1944–1964 [15]), because dynamics are predominantly annual, extreme deviations from this pattern and corresponding biases are relatively unlikely.

### 2.3. Critical community size

Because population size is a key determinant of stochastic extinction for strongly immunizing infections, population size is negatively correlated with the number of fade-outs (or proportion of zeros) in the time-series of incidence [15,23]. The point where this line intercepts with zero provides an indication of the critical community size, or population size below which the infection is subject to stochastic fade-outs. Because under-reporting could lead to apparent fade-outs where there are none, we define fade-outs as corresponding to a month with zero reported cases.

### 2.4. Spatial dynamics: connectivity

To quantify spatial dynamics, we can define a spatial coupling parameter that measures how tightly each region is linked to the metapopulation [24]. During fade-outs, in location *j*, the probability that no epidemic results following a spatial contact is 1/(1 + *β_s_S_t_*_,*j*_), and conversely, a new epidemic is sparked according to the time-varying hazard:2.5
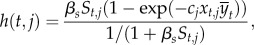


where *c_j_* is a parameter that describes the coupling of community *j* to the regional metapopulation; *x_t_*_,*j*_ is the local proportion of susceptibility (*S_t_*_,*j*_/*N_t_*_,*j*_,) and 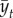 is the probability that a non-local individual is infectious 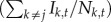. Methods from survival analysis can be used to estimate *c_j_* for every location [20,24].

This analysis can be extended to identify a parametric form for connectivity such as specified by the gravity model [25] for which contacts between locations *j* and *i* is given by2.6
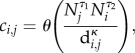
to capture the fact that traffic to a particular district is expected to increase in a generalized bilinear fashion with recipient and donor population sizes (*N_j_* and *N_i_*) but decrease with some measure of their separating distances, *d_i,j_* (which might reflect Euclidean distances, road distance, cost of travel, etc., as described above). The *θ*-parameter scales the overall mobility and the exponents *τ*_1_, *τ*_2_ and *ρ* controls the topology of the spatial network. Within this more spatially resolved model of coupling, new epidemics will be sparked according to the refined version of equation (2.5)2.7
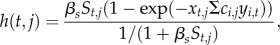


where *y_i_*_,*t*_ is the probability that an individual from patch *i* is infectious (*I_i_*_,*t*_/*N_i_*_,*t*_). To counter biases due to under-reporting, we restricted this analysis to time-points where four weeks had passed with no reported rubella incidence.

### 2.5. Simulating the metapopulation dynamics of rubella in South Africa

Using the magnitude of seasonality, and connectivity between locations, we developed a simulation model of rubella in South Africa (see the electronic supplementary material, appendix S1). The key element of the model is a matrix that, at every time-step, defines transition from every possible epidemiological stage (e.g. infected, susceptible and recovered) and age combination to every other epidemiological stage and age combination, following methods developed in [26]. This was extended to also capture spatial dynamics where the number of immigrants was specified according to connectivity estimates described above. A key element of the model is the pattern of contacts and transmission over age. This can be captured by a Who-Acquires-Infection-From-Whom (WAIFW) matrix. Every cell of the matrix captures the strength of contact between the age classes represented by each row and column. We evaluated two different WAIFW structures (i) a model of age contacts fitted using a smooth surface [27], and (ii) the empirically derived POLYMOD matrix [28]; and evaluated their validity using approaches developed by Rohani *et al*. [29]. We used the framework to explore the impact of vaccination on the CRS burden, obtained by combining the fertility profile of South Africa with the simulated age profile of incidence (see the electronic supplementary material, appendix S1).

To identify how spatial heterogeneity in vaccination might affect age-incidence for rubella, and consequently the CRS burden, we generated vaccination profiles reflecting the same overall mean coverage, but a range of different distributions across space using a beta-binomial distribution. Given the importance of population size for patterns of human movement implied in the connectivity analyses (see below), we particularly wished to explore the impact of the degree to which vaccination coverage was correlated with population size. We refer to the strength of this correlation as the degree of polarity. To implement a range of polarities, we used ‘fuzzy ordering’. This involves generating normal deviates around the coverage values obtained from the beta-binomial (see above). Values of coverage for a particular population size are then paired up according to the ordering of these normal deviates. Lower variances used to derive deviates will result in tighter correlations between population size and coverage; the opposite results from high variances. In this way, we can create a gradient ranging from low to high ‘polarity’. The resulting vector of vaccination coverage will have slightly different levels of population scale coverage, given the distribution of coverages among populations. We therefore adjusted the resulting vector of vaccination probabilities to have the same mean at the scale of the population (set to 0.8) by multiplying the coverage values by a constant. We then explored the effect of the range of vaccination polarity scenarios on the CRS burden of the population as a whole, and within districts.

To evaluate the prospects of introduction of rubella-containing vaccine in South Africa, we implemented vaccination scenarios reflecting actual reported coverage levels for measles across districts using the data described above. To be conservative, we considered only the first dose of measles containing vaccine, and did not model supplementary immunization campaigns. Further, to consider the ‘worst case scenario’ and to reflect the discrepancy between country-reported values and UNICEF-WHO values at the national scale (UNICEF-WHO adjusted values tend to be rather lower than national reports [30]), we used simulations with both reported estimates of coverage, and by reducing all estimates of coverage by 15 per cent.

## 3. Results

**Figure 1.**
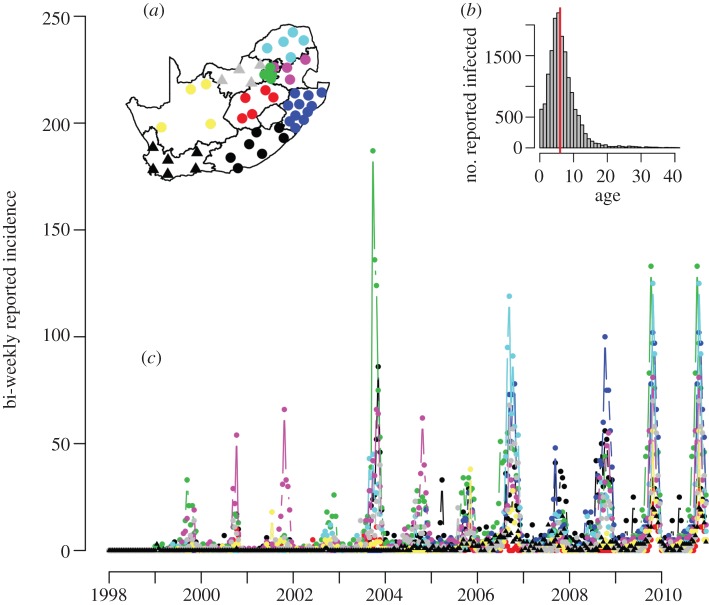
Raw time-series of reported rubella incidence aggregated into bi-weeks across 13 years in South Africa for nine provinces (corresponding to 52 districts). Insets (*a*) corresponding districts (points) and provinces (contours, colours) of South Africa; colours on the map correspond to colours in the time-series (ECP, black for the Eastern Cape Province; FSP, red for the Free State Province; GAP, green for Gauteng Province; KZP, blue for KwaZulu-Natal province; LPP, turquoise for Limpopo province; MPP, pink for Mpumalanga; NCP, yellow for the Northern Cape Province; NWP, grey for the northwest province; WCP,  black with triangles symbols for the Western Cape Province; and (*b*) the distribution of rubella incidence over age for the entire country, median of six shown as a vertical line.

The data consist of weekly time-series of reported rubella incidence from 1998 to 2010, stratified by the 52 districts of South Africa (figure 1*a*) and by age (figure 1*b* and table 1). The dataset report on a total of 16 466 cases. The country-wide median age of infection was 6 years (figure 1*b*), and within any single week the case numbers ranged from zero to 90 reported cases (figure 1*a*). Country-wide outbreaks follow a predominantly annual pattern, but local dynamics are more variable.

**Table 1.** Province characteristics. Average age of infection is the mean reported age from incidence reports from each province across the entire time-series; birth rates are taken as described above; and under-reporting is obtained via susceptible reconstruction from the TSIR (see §2) and reflects the proportion of individuals that are reported in every time-step out of all the individuals that were infected with rubella. Abbreviations refer to ECP, Eastern Cape; FSP, Free State; GAP, Gauteng; KZP, KwaZulu-Natal; LPP, Limpopo; MPP, Mupumalanga; NCP, Northern Cape; NWP, North West; WCP, Western Cape. Note that the average age of infection is likely to be an underestimate, owing to biases from measles-targeted sampling; the *R*_0_ estimate may be correspondingly high; in the associated estimation, *G* refers to the inverse of the birth rate and *A* is the average age of infection.

	ECP	FSP	GAP	KZP	LPP	MPP	NCP	NWP	WCP
average age of infection, A	7.0	7.1	5.9	6.0	7.0	7.0	8.0	7.0	6.0
birth rate per 1000 per year, B	22.4	20.5	20.3	23.0	24.2	23.5	21.1	22.76	22.09
estimate of *R*_0_, as 1 + G/A, where *G* = 1000/B	7.4	7.9	9.3	8.2	6.9	7.1	6.9	7.3	8.5
average under-reporting	0.00045	0.00026	0.00035	0.00031	0.00031	0.00036	0.00038	0.00039	0.00038

### 3.1. The time-series susceptible–infected–recovered

**Figure 2.**
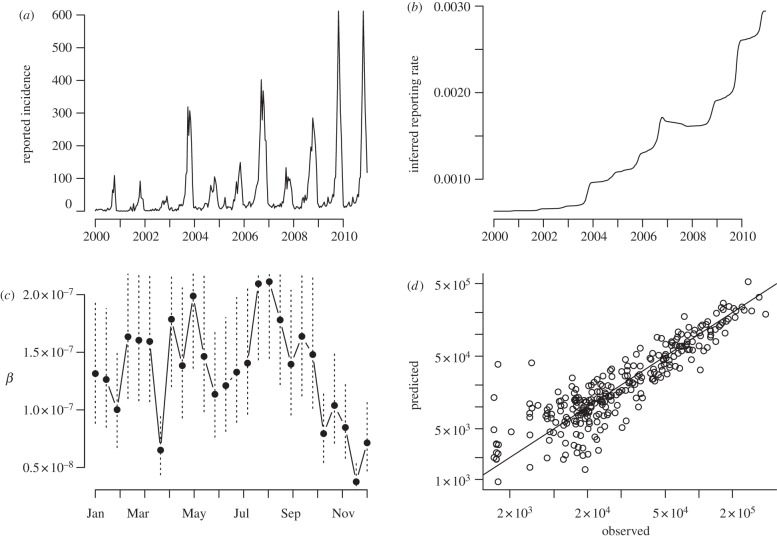
TSIR estimates of seasonal transmission rates taken from data starting in 2000 across the entire country showing (*a*) the full time-series used for estimation; (*b*) the estimated under-reporting rate through time from the smooth spline on susceptible reconstruction; (*c*) the pattern of transmission over the season, with standard errors shown as vertical dashed lines; and (*d*) the relationship between observed and expected numbers of total infected individuals in each bi-week for the fitted model; where the observed is obtained by dividing through the reported numbers by the reporting rate (figure 2*c*). The line indicates where *x* = *y*. The corresponding estimate of R_0_ is taken as 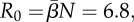 where 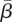 is the mean transmission rate.

We applied a smoothing spline with 6 degrees of freedom to patterns of birth and incidence taken across the entire country (figure 2*a*) for susceptible reconstruction for years starting in 2000. This indicates an increase in reporting over the time-course of the data (figure 2*b*), with a steep increase during 2009–2010 when a measles epidemic occurred. We then identified the starting proportion susceptible and seasonal pattern of transmission using profile likelihood. As detailed in §2, we fixed *α* at the consensus estimate of 0.97 [15,18,20] before fitting the TSIR. The estimated seasonal pattern of transmission reflects the timing of school holidays (figure 2*c*), with low transmission during the school summer vacations in South Africa (usually around four weeks including the 25 December and 1 January). The TSIR model provides a good fit to the short-term dynamics of the infection (figure 2*d*). We also used susceptible reconstruction in each province to estimate province-specific reporting rates (equation (2.2)), given expectations of heterogeneity among districts in reporting rates.

### 3.2. Spatial dynamics

To estimate connectivity, we used the country-wide under-reporting rate to reconstruct the susceptible profile of each district, as province-specific estimates of under-reporting did not capture the expected increase in reporting rate over time (figure 2*b*) and detectable differences between provinces were relatively small (table 1). The estimated value of regional coupling (equation 2.5, binomial log likelihood of −2136 for 52 parameters on *N* = 9875 data points) significantly increased with population size (see the electronic supplementary material, figure S1), and this relationship explained a considerable amount of the variance (*r*^2^ = 0.32). For the parametric estimate of coupling, a comparison of the binomial log likelihood resulting from equation (2.7) indicates that for both Euclidean distance models and ‘cost’ models for *d_i,j_*, the *κ* coefficient is so small that the denominator is basically 1, indicating that differences in connectedness are predominantly explained by population size. For *N* = 9875 data points, with a log likelihood of −2635, parameters are *θ* = 7.9 × 10^–12^ (CI: 1.9 × 10^–13^, 3.5 × 10^–10^, confidence interval obtained by inverting the hessian [31]), *τ*_1_ = 1.4 (CI: 1.2, 1.5), *τ*_2_ = 1.1 (CI: 0.9, 1.4), *κ* = 0 (CI: 0, 0), for both the cost and distance models; identical values are obtained if the denominator is dropped.

### 3.3. The critical community size

**Figure 3.**
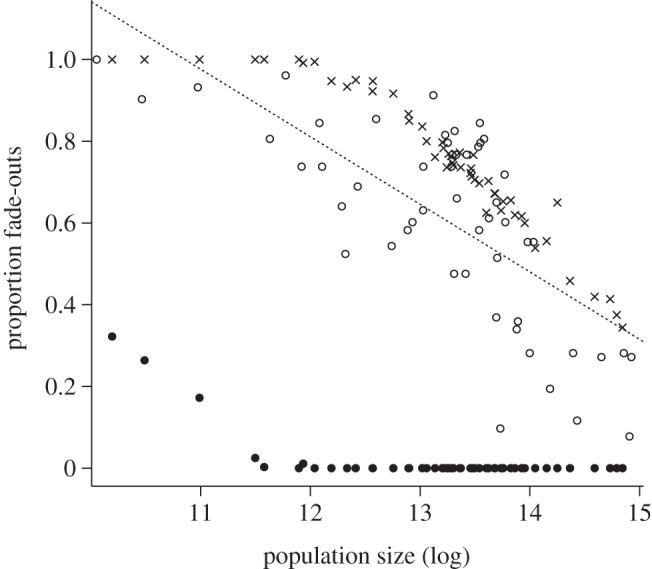
Comparison of observed and simulated proportion of fade-outs in each of the 52 districts, showing both directly observed fade-outs in the simulation (filled circles), and fade-outs observed (open circles) if the definition of the extinction threshold is corrected by the reporting rate (crosses), e.g. a fade-out is recorded for time-steps where the product of simulated incidence and 0.0015 is less than 1 (0.0015 being the average reporting rate; figure 2*b*). A linear regression fitted to the observed data has the form *y* = 2.75 – 0.16*x*, *p* < 0.001, *r*^2^ = 0.50. The line intersects zero at a population size greater than exp(16), implying a large CCS. The simulated data indicate a CCS of approximately exp(12), or 170 000, which is much lower.

The proportion of zeros for each district in the time-series is tightly negatively linked to log district population size (*F*_1,50_ = 49.1, *p* < 0.001), suggesting that the CCS is larger than a million (figure 3). However, given the risk of under-reporting, this is likely to somewhat overestimate the CCS. To assess this, using parameter estimates described above, we established a model for age-structured rubella dynamics across the 52 districts in South Africa. We evaluated the model by comparing the predicted incidence, age profile of infection and profile of fade-outs with the observed (see the electronic supplementary material, figure S2–S4; figure 3). The pattern of incidence and its age profile are in accord with the data; the pattern of the profile of fade-outs in the simulations relative to the observed implies that on the basis of parameters available here, the CCS for rubella is broadly overestimated using the method of proportion of fade-outs over time; the model prediction yields a value closer to 100 000 than 1 000 000.

### 3.4. Impact of introduction of rubella vaccine on the congenital rubella syndrome burden

**Figure 4.**
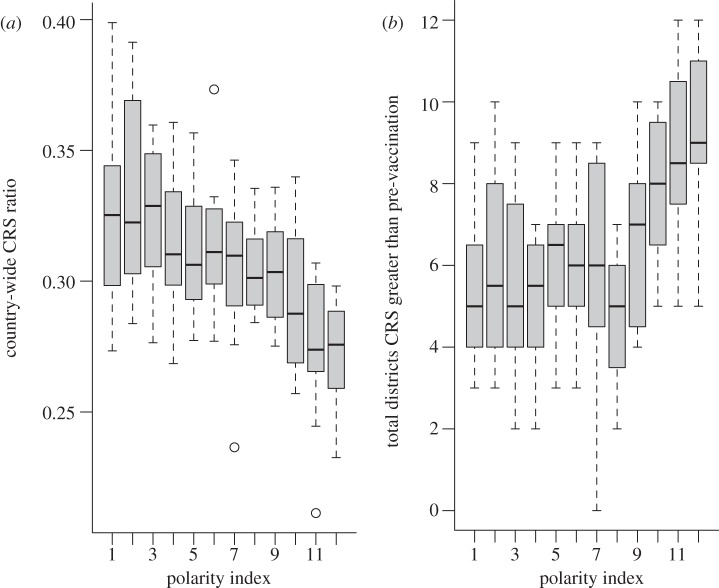
(*a*) Country-wide CRS ratio per 1000 pregnancies over 30 years before and after vaccination introduced with a coverage of 80% at the country scale across 12 simulations for increasing levels of polarity (*x*-axis; which reflects the magnitude of the correlation between vaccine coverage and district population size); and (*b*) for the same simulations, the number of districts for which the CRS burden per 1000 pregnancies increases following the introduction of vaccination. The reverse pattern is observed at the global and local scales.

Using the structured model described above, we implemented a range of vaccination coverage scenarios, to compare the CRS burden across a range of polarities of vaccination coverage in situations where the country-wide coverage level is retained constant (see the electronic supplementary material, figure S5). This analysis indicates that high polarity may have opposite effects at the global and local scale. The country-wide CRS ratio is lowest for simulations that correspond to the highest polarity in vaccination coverage (figure 4*a*), while the number of districts with an increase in the CRS burden is highest (figure 4*b*). This indicates that where the correlation between coverage and district population size is high (corresponding the high polarity), although the population of South Africa as a whole may experience a decrease in the relative burden of CRS, particular districts may experience an increase. This occurs because in high-polarity simulations the strong correlation between coverage and district size means that many of the large districts that are sources of infected immigrants are vaccinated. Consequently, the number of circulating infected immigrants is lower. This means that there can be a longer wait before a fade-out ends in the districts with smaller population sizes that are below the CCS. Because this waiting time enables susceptible individuals to age into childbearing years, these districts have a much higher CRS burden than observed in the absence of vaccination.

**Figure 5.**
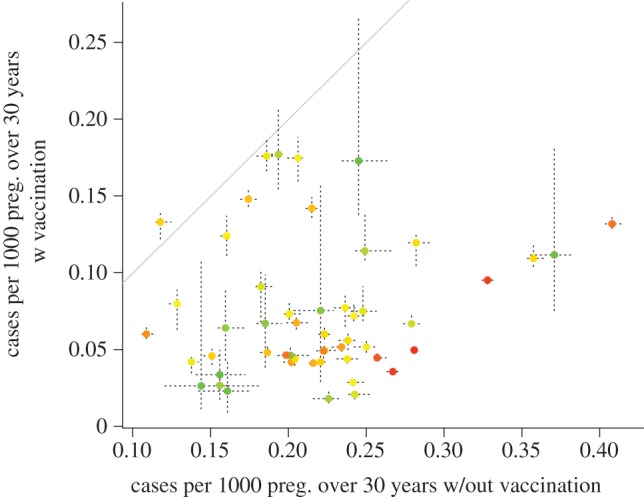
Predicted burden per 1000 pregnancies for each district over 30 years in an unvaccinated population (*x*-axis) versus a vaccinated population (*y*-axis), vaccinated following the most recent data on vaccination coverage across districts in South Africa, adjusted by subtracting 15% (see §2). Points indicate medians across 10 simulations, coloured to reflect population size (red is the largest population size); and dashed horizontal and vertical lines indicate the ranges across the 10 simulations. Note that larger districts have smaller variation across the 10 simulations as they are less prone to stochastic extinction. The *y* range is almost entirely below the *y* = *x* line (grey), indicating no evidence for an increase in the CRS burden following vaccination, except for DC26 (which experienced 63% coverage in 2000). The country-wide burden over 30 years is 6201 CRS cases (ranging from 6167 to 6220) in the absence of vaccination, versus 1930 (ranging from 1912 to 1966) with adjusted reported coverage.

The simulations of the observed coverage for South Africa indicate that if the current estimates reflect future coverage, the chances of an increase in the burden of CRS per 1000 live births across 30 years are relatively low (figure 5, all points bar one are below the *y* = *x* line). We explored robustness of the conclusion that even with coverage as low as 65 per cent (the average fitted in figure 5) the CRS burden was reduced, via a range of sensitivity analyses. Repeating the analysis shown in figure 5 using a fitted WAIFW (see the electronic supplementary material, figure S2, second row) rather than the polymod WAIFW resulted in no increase in the CRS burden at the national scale, indicating robustness in the pattern of transmission over age (see the electronic supplementary material, figure S6). Increasing transmission as high as *R*_0_ = 12 with the polymod WAIFW did lead to an increase in the CRS burden in a subset of districts over 30 years, but the country-wide outcome remained a reduction in the CRS burden (see the electronic supplementary material, figure S7).

## 4. Discussion

While the burden of CRS may be small relative to the overall disease burden of children in certain regions, the low cost of the rubella vaccine, the relatively low transmission rate of the infection compared with measles and the ability to deliver vaccine without administering an extra shot mean that it may be one of the diseases most effectively tackled. Conversely, once immunization against rubella is implemented, ceasing vaccination is likely to result in a considerably increased CRS burden; so long-term maintenance of coverage must be assured.

The classic concern for rubella vaccination has been that insufficient vaccination coverage of children may lead to an increase in the burden of CRS because of an increase in the average age of infection, insufficiently offset by reduced incidence. However, evidence, including high average age of rubella infection [20,32,33], in many countries suggests that rubella may frequently have a low *R*_0_ (although see [34]). In this situation, reducing incidence requires relatively lower coverage (particularly if the birth rate is not too high [6,35]), and problems may rather emerge from stochastic dynamics in a metapopulation and waiting times following local extinction before re-introduction [20]. Given an average age of infection for rubella in South Africa suggesting that *R*_0_ is not excessively high (table 1, even in the face of a likely downward bias due to sampling targeted at measles that classically has a much lower age of infection), and the relatively low birth rate (around 20 per 1000 per year https://www.international.ipums.org/international/), this was the focus of our analysis here.

We first characterized seasonal dynamics and the metapopulation structure of rubella transmission in South Africa. Susceptible reconstruction suggested changes in reporting rates compatible with the occurrence of a measles outbreak from 2003 to 2005 [36] and a larger outbreak in 2009 and 2010 [37]. Seasonal dynamics followed school terms, as frequently observed for immunizing childhood infections [19] including rubella [20,33] and district population size proved to be the key determinant of movement between districts (as measured via the duration of rubella fade-outs, see above), with larger populations producing both more infected emigrants and attracting more infected immigrants (in line with previous work [24,38]). This conclusion may be affected by the fact that spatial variation in reporting rates is likely, with a higher index for reporting and testing in some provinces (e.g. Gauteng, Western Cape and KwaZulu-Natal and perhaps Northern Cape) than in others (Limpopo, Mpumalanga, North West, Eastern Cape), a pattern likely to be correlated to some extent with urbanization and population density; although this did not emerge strongly in estimates of coverage obtained via susceptible reconstruction (table 1). Incorporating Euclidean distance between districts or ‘cost of travel’ did not improve the model, but this might be partly because the spatial scale of case data available was too coarse relative to the range of actual travel decisions (districts often cover large areas, and many settlements, thus the representation of each with distances or costs of travel between single district centroids is likely to be an oversimplification). This observation brings to the fore an important caveat relative to the broader predictions of our model: if district population sizes do not actually reflect population sizes relevant to epidemiological dynamics, then predictions relative to changing CRS burdens across the region in response to vaccination will be affected. For all our model predictions, caveats linked to heterogeneity in reporting rates should also be considered.

The missing element for developing a simulation of rubella is then the structure of transmission over age. We used two approaches (i) we fitted a smooth WAIFW to the observed age-incidence data combined with data on the demographic structure of the population (see the electronic supplementary material, appendix S2) and (ii) we used the average WAIFW indicated by diary studies across Europe (both shown in the electronic supplementary material, figure S2). The former cannot capture the complexity of changing contacts between adults and children, but conversely, it is not clear that the latter is appropriate in the social and cultural context of South Africa. However, both provided similar qualitative conclusions, and are likely between them to capture a large part of the possible range of transmission structures. Empirical surveys that could ascertain the validity of these approaches are desirable, however; and again, model predictions will be vulnerable to misspecification of contacts over age.

Combining parameters obtained from the rubella incidence data, with the chosen transmission WAIFW, we developed a model to characterize the effect of vaccination on the burden of CRS. Overall, our results indicate that a global reduction in infectious individuals moving between districts may increase the burden in smaller populations below the CCS if these are unvaccinated, because of longer waiting times following extinction (figure 4), making the interaction between population movement, coverage and the CCS a key equity question. However, the current vaccination coverage results overall in a reduction in the burden of CRS in South Africa over a 30 year time horizon (figure 5). The absence of a relationship between district population size and reported coverage for measles (e.g. in 2000, the correlation between vaccine coverage and log population size was *ρ* = 0.05, d.f. = 52, *p* > 0.1; similar results in other years) and relatively low variance in coverage (*σ*^2^ = 0.03 across all years; and that or lower for each individual year) is likely to be a key contributing factor. It is, however, key to note that this prediction is vulnerable to errors of model misspecification, in particular the relevance of the spatial scale of the data may affect conclusions, but uncertainties in reporting and questions relative to the age-structure of transmission will also play a role; and our estimate of CRS incidence of approximately 200 CRS cases per year (figure 5) may consequently be either an over- or underestimate (previous analyses based on sero-prevalence suggest a value of around 600 [39]).

An interesting point revealed by the simulations is that it is apparent that the CCS may have been overestimated in previous work [33,40), given under-reporting (figure 3); in reality the CCS for rubella might be much closer to that of measles (see the electronic supplementary material, figure S8). Although, theory predicts a slightly larger value for rubella given that its *R*_0_ is generally lower than that of measles [41], this theoretical analysis did not incorporate seasonality in transmission, and troughs between major outbreaks due to seasonality are likely to determine extinction probability and increase with the *R*_0_ of the infection (see the electronic supplementary material, figure S8).

**Figure 6.**
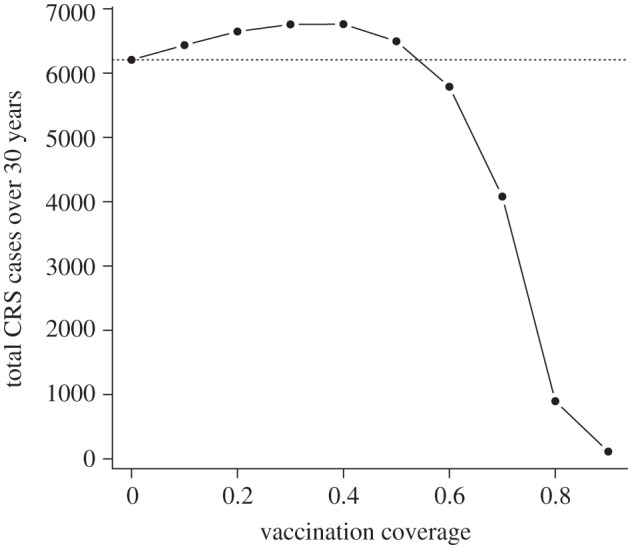
Assuming homogeneous coverage over the whole country, and using the POLYMOD WAIFW, predicted CRS burden over 30 years for the whole country, for values of coverage between 0 and 0.9 showing the median and range of three simulations for each point. This indicates that in the simple case of relatively homogeneous coverage across the country, and assuming no correlation between routine vaccination (i.e. no consistently under-served population) the burden of CRS will be increased for small amounts of private-sector coverage; but reduced for levels of coverage greater than 50%.

In the absence of broad stochastic effects on age-structure driven by extinction and re-colonization linked to the CCS, one might expect the response of rubella in South Africa to vaccination to be in line with previous work that suggested the 80 per cent cut-off rule [42–45]. However, our predictions are in line with more recent work suggesting that with a birth rate around 20 per 1000 per year and an *R*_0_ around 6, increases in CRS over a 30 year time horizon are unlikely (although, again, model misspecification remains a potential concern). In this context, an important public health dimension of the introduction of the rubella vaccine is the degree to which it is prevalent in the private sector [45], i.e. for individuals who receive for example MMR vaccination in an unregulated market where all vaccines are available [39]. Assuming no spatial heterogeneity in coverage, our model suggests that such private-sector vaccination with levels of coverage between 20 and 30 per cent should be particular cause for concern (figure 6). Previous analyses suggest coverage of around 15–20% in such unregulated markets [39].

To conclude, data available for South Africa shed light on basic aspects of rubella epidemiology (CCS, connectivity, seasonality), but also highlight areas of consideration in a public health setting, including metapopulation-induced changes in age-incidence, which can lead to public health equity issues. The methods we have developed could be use to explore the impact of heterogeneous vaccination in other connectivity contexts and for other infections with an age-specific impact, such as mumps. Interestingly, our model predictions are broadly positive relative to the introduction of routine rubella vaccination in South Africa, despite the relatively low measles vaccine coverage levels explored, with possible relevance to a number of countries in the region. Of course, these conclusions rest on the model assumptions (detailed above), and the data available. Model misspecification is always a risk, and key areas for future research include further detail on the age-transmission profile of rubella in developing and middle-income country settings.
